# Case Report: A Chronological Combination Treatment of Icotinib, Osimertinib, and Crizotinib on Lung Adenocarcinoma Guided by Serial Genetic Tests of Circulating Tumor DNA and Sediment Cell Genomic DNA From Pleural Effusion

**DOI:** 10.3389/fonc.2020.561341

**Published:** 2020-10-23

**Authors:** Zhihua Miao, Tianhao Mu, Longying Liu, Jingjie Rao, Min Jin, Zhizheng Wang, Hui Wang, Chao Lu, Xiaolin Gong, Dongcai Zheng, Daoming Zheng, Xin Li, Yingmei Li, Shifu Chen, Xinhua Shu

**Affiliations:** ^1^Shangrao Guangxin Traditional Chinese Medicine Hospital, Shangrao, China; ^2^HaploX Biotechnology, Shenzhen, China

**Keywords:** lung adenocarcinoma, serial genetic monitoring, EGFR mutation, MET amplification, ctDNA and PE-sDNA

## Abstract

Precision medicine has been getting more attention in lung cancer treatment. Here, we report an unusual case of a 71-year-old Chinese male patient with poorly differentiated lung adenocarcinoma with lymph node metastasis. A 5 years' treatment history of this patient is reported. By serial genetic tests of circulating tumor DNA (ctDNA) from peripheral blood and sediment cell genomic DNA (PE-sDNA) from pleural effusion, a novel chronological combination treatment of icotinib, osimertinib, and crizotinib was adopted for the present genetic mutations, including *EGFR* exon 19 deletion, *EGFR* p.T790M, and *MET* amplification.

## Background

Lung adenocarcinoma is the most common subtype of non-small cell lung cancer (NSCLC), which accounts for more than 500,000 deaths per year (1). Fortunately, dozens of drugs can be used for the treatment of patients with certain oncogenic alterations, including mutations of *EGFR, ALK, ROS1*, and so on (2). In recent years, precision medicine based on next-generation sequencing (NGS) has been widely used as a companion of targeted drug treatment (3). It is well-recognized that cancer cells evolve and exhibit different sets of genetic mutations as the disease progresses. Hence, understanding genetic mutations at different stages of disease progression is essential for precision medicine. However, obtaining sufficient tumor materials for molecular profiling is still challenging in current practice. Liquid biopsy based on circulating tumor DNA (ctDNA) has been used as a novel minimally invasive method to detect the genetic alterations of patients (4). In addition to ctDNA, pleural effusion (PE) is developed in about 30% of NSCLC and can be considered as an alternative minimally invasive approach to detect tumor driver mutation. PE containing both tumor-derived cell-free DNA (cfDNA) and sediment cell genomic DNA (sDNA) has been applied to detect EGFR mutations by ARMS PCR, digital PCR, and Sanger sequencing in the previous studies (5–9). However, investigation of large-scale genomic profiling by sediment cell genomic DNA (PE-sDNA) from pleural effusion has not been fully understood. Here, we report a Chinese patient with lung adenocarcinoma who benefited from a chronological combination treatment of icotinib, osimertinib, and crizotinib guided by serial genetic tests of circulating tumor DNA (ctDNA) from peripheral blood and sediment cell genomic DNA (PE-sDNA) from pleural effusion.

## Case Presentation

**Figure 1:**
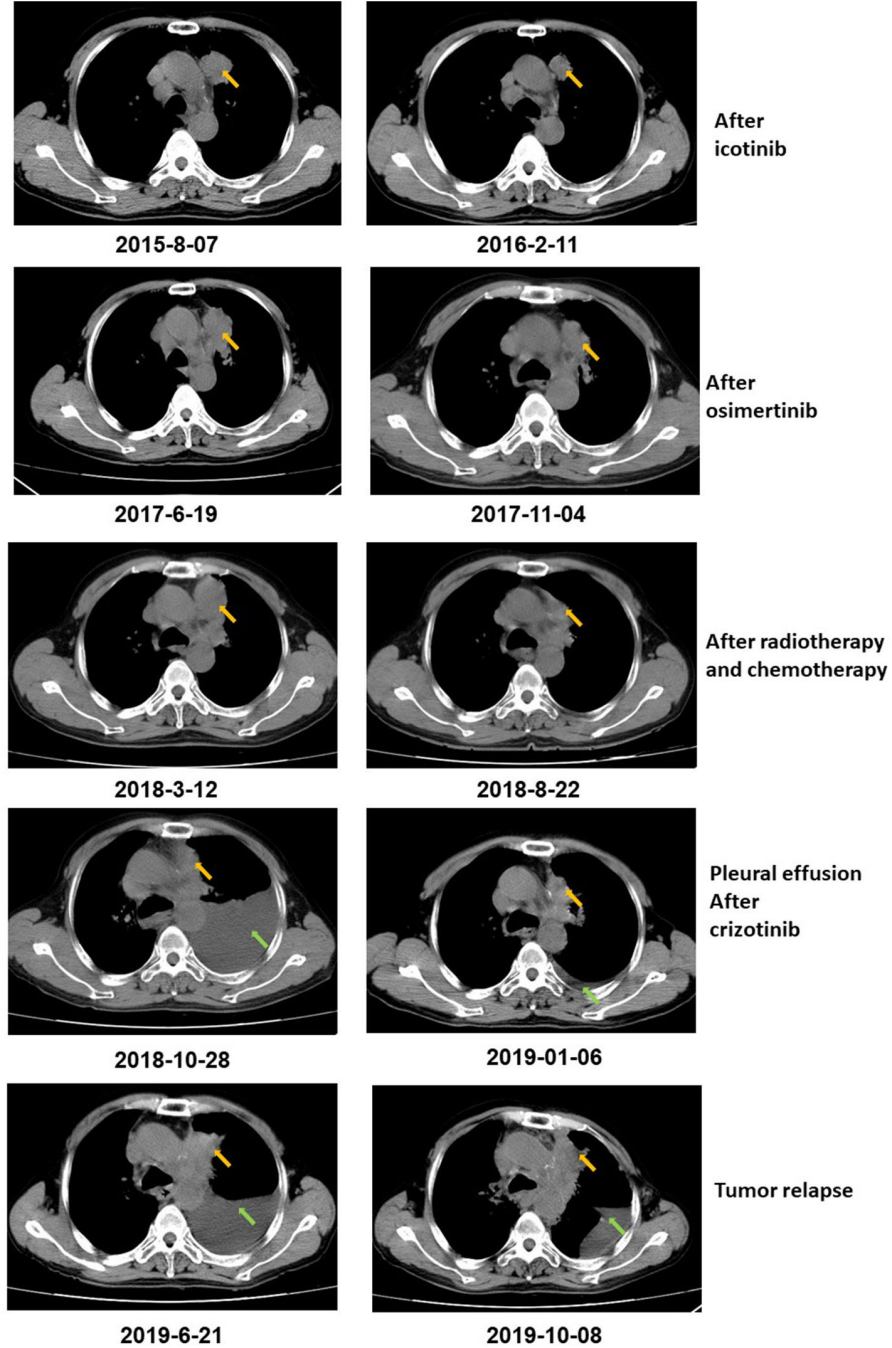
CT scan showed that the tumor size decreased after icotinib treatment, osimertinib treatment, and radiotherapy and chemotherapy, respectively. After icotinib treatment, the tumor size (yellow arrow) decreased from 39 mm*36 mm to 39 mm*27 mm in February 2016. After icotinib-resistance and osimertinib treatment, the tumor size (yellow arrow) decreased from 42 mm*34 mm to 40 mm*22 mm in November 2017. After radiotherapy and chemotherapy during March 2018 to June 2018, the tumor size decreased from 49 mm*32 mm to 41 mm*20 mm. After October 2018, pleural effusion (green arrows) was developed in the patient and extracted. The patient achieved a stable disease (SD) during crizotinib treatment in June 2019, compared with October 2018, with tumor mass measuring from 36 mm*30 mm to 36 mm*27 mm.

A 71-year-old male patient without a family history was diagnosed with poorly differentiated lung adenocarcinoma with lymph node metastasis (Figure 1). The patient was treated by a chronological combination of icotinib, osimertinib, and crizotinib with a series of genetic sequencing. A total of 5 years' treatment details and other clinical data were collected. Targeted sequencing with a panel of 605 cancer-related genes (Supplementary Information 1) was performed at multiple timepoints during the treatment. Circulating tumor DNA (ctDNA) from peripheral blood and sediment cell genomic DNA (PE-sDNA) from pleural effusion were sequenced.

**Figure 2:**
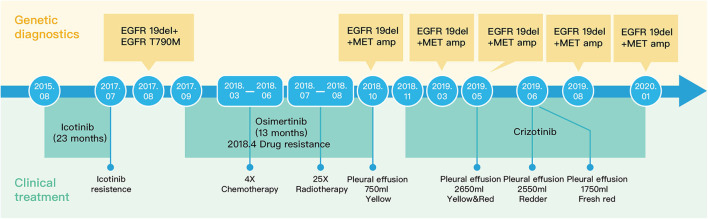
Schematic diagram of the treatment of the patient.

**Figure 3:**
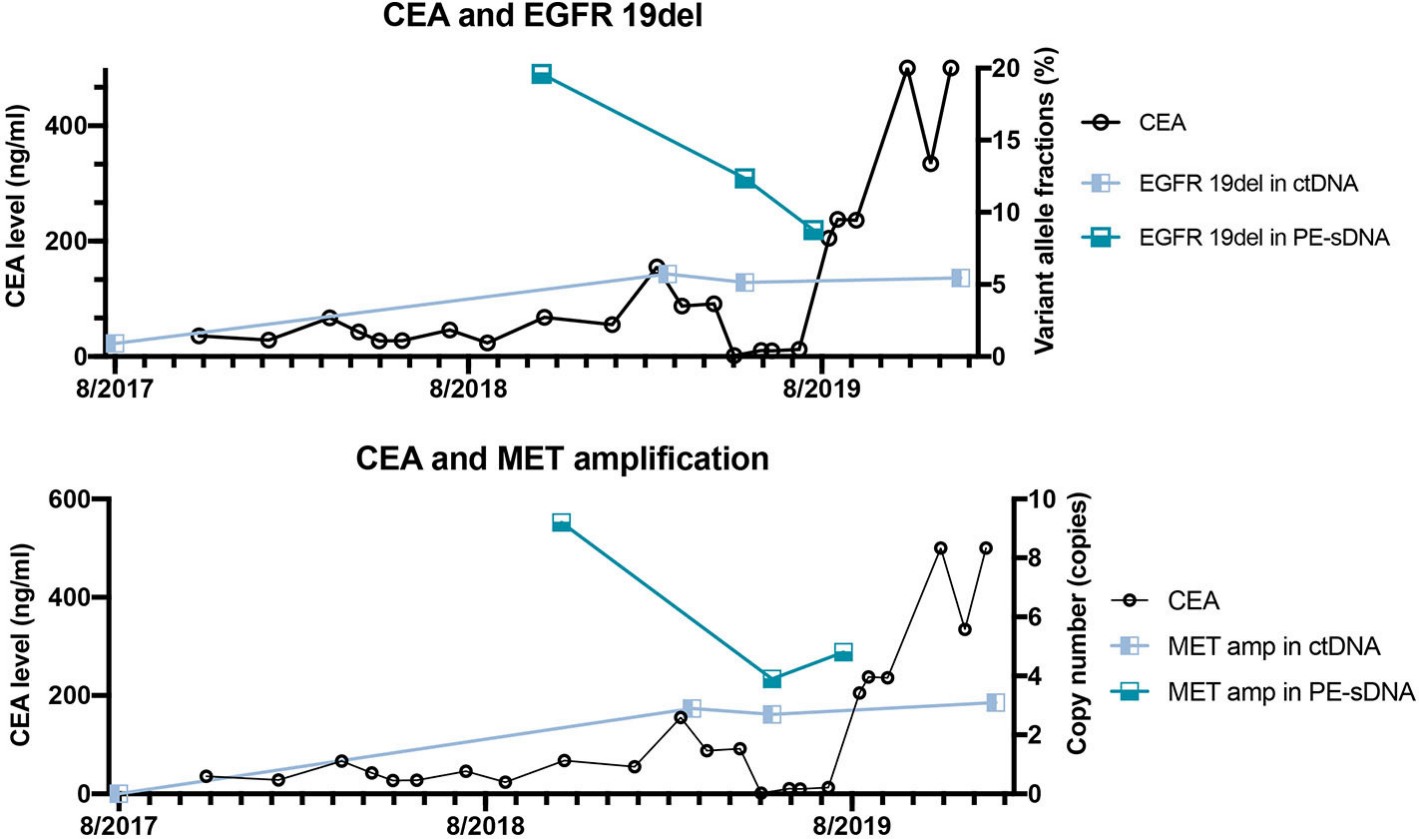
The correlation between dynamic variant allele fractions/copy numbers of mutated genes and the level of carcinoembryonic antigen (CEA) of the patient over 2 years. The left Y axis represents the CEA level. The right Y axis represents the variant allele fractions of *EGFR* 19del (upper panel) or the copy number of *MET* amplification (lower panel). The X axis represents the date of detections.

The patient was diagnosed with lung adenocarcinoma at stage T3N3M0IIIC in August 2015. The tumor occurred in the left upper lobe and was identified as poorly differentiated adenocarcinoma with metastasis in the left supraclavicular lymph node. The first CT scan showed a tumor mass measuring 39 mm*36 mm at baseline. EGFR mutation was confirmed by immunohistochemistry assay. The patient started to receive icotinib (first-generation EGFR-TKI) therapy for 23 months since August 2015. The tumor mass decreased to 29 mm*27 mm and the patient achieved stable disease (SD) status in February 2016. Drug resistance was acquired with progressive disease (PD) in the patient during icotinib treatment afterwards. A CT scan showed a tumor mass measuring 42 mm*34 mm in June 2017. The first genetic test of ctDNA showed *EGFR* exon 19 deletion and T790M mutations (variant allele fractions (VAFs) of *EGFR* 19del and T790M were 0.91 and 0.35%, respectively) in August 2017 after icotinib-resistance (Figure 2, Table 1). The strategy of targeted drug treatment changed from icotinib to osimertinib in September 2017 and lasted for 14 months. The patient achieved stable disease (SD) and another CT scan with a tumor mass measuring 40 mm*22 mm in November 2017 demonstrated that osimertinib worked well and the tumor mass decreased (Figure 1). However, the patient presented progressive disease (PD) and osimertinib-resistance after 8 months' treatment. Next, the patient received 4 cycles of chemotherapies (pemetrexed 500 mg/m^2^ plus cisplatin 75 mg/m^2^) and 25 cycles of radiotherapies (50 Gy) companied with osimertinib from March 2018 to August 2018. After radiotherapies and chemotherapies, the tumor mass decreased from 49 mm*32 mm to 41 mm*20 mm, and the patient achieved stable disease (SD). In October 2018, the patient developed pleural effusion, and 750 ml of pleural effusion was extracted and analyzed. *EGFR* 19del (19.96%), *TP53* c.994-1G>C (27.36%), *FGF23* p.L224^*^ (18.57%) and *MET* amplification (9.2 copies) were identified, while *EGFR* T790M was negative in pleural effusion. Crizotinib was adopted for the patient against *MET* amplification in November 2018. Another three extractions of pleural effusion from the patient and a continuous series of genetic tests were performed to monitor the genetic alterations until the death of the patient (February 2020). The patient achieved stable disease (SD) during crizotinib treatment in June 2019, compared with October 2018, with tumor mass measuring from 36 mm*30 mm to 36 mm*27 mm. *EGFR* 19del and *MET* amplification were present during the whole treatment course of crizotinib, without the presence of *EGFR* T790M. *TP53* c.994-1G>C and *FGF23* p.L224^*^ were also exhibited in the patient. We find that VAFs and copy numbers of mutated genes in ctDNA were lower than those in PE-sDNA among the whole genetic tests of the patients (Table 1). Especially on May 24th, 2019, when both PE-sDNA and ctDNA were detected, the same mutations were presented by both PE-sDNA and ctDNA, while VAFs and copy numbers of all detected mutations by PE-sDNA were higher than those by ctDNA (Table 1). This implies that PE-sDNA contains a higher proportion of tumor cells compared with ctDNA. In another aspect, the comparison of two separate genetic tests can be made in the same sample type (either ctDNA or PE-sDNA). After taking crizotinib, *MET* amplification in PE-sDNA dramatically decreased from 9.2 copies to 3.8 copies in May 2019 and increased to 4.6 copies in August 2019. *MET* amplification in ctDNA showed a similar changing pattern as PE-sDNA from March 2019 to January 2020. Dynamic VAFs of other mutations, including *EGFR* 19del, *TP53* c.994-1G>C, and *FGF23* p.L224^*^, generally showed a similar pattern with *MET* amplification, which was also consistent with changes of the expression level of carcinoembryonic antigen (CEA) in the patient (Figure 3, Supplementary Information 2). These results implied that the tumor was alleviated shortly after receiving crizotinib and relapsed later, which is consistent with increased tumor mass measuring 48 mm*30 mm by CT scan in October 2019.

**Table 1:** Genetic mutations of multiple genetic tests.

**Gene**	**Mutation**	**2017.8.11**	**2018.10.26**	**2019.3.5**	**2019.5.24**	**2019.8.3**	**2020.1.2**
		**ctDNA**	**PE-sDNA**	**ctDNA**	**PE-sDNA/ct-DNA**	**PE-sDNA**	**ctDNA**
EGFR	p.E746_A750del	0.91%	19.96%	5.72%	12.36%/5.15%	8.77%	5.45%
EGFR	p.T790M	0.35%	–	–	–	–	–
MET	Amplification	–	9.2 copy	2.9 copy	3.8/2.7 copy	4.6 copy	3.1 copy
TP53	c.994-1G>C	–	27.36%	1.80%	5.25%/1.23%	7.53%	3.93%
FGF23	p.L224^*^	–	18.57%	1.12%	4.09%/1.09%	5.11%	3.32%
TMB	–	–	4.58 Muts/Mb	3.82 Muts/Mb	4.58/4.58 Muts/Mb	3.82 Muts/Mb	4.58 Muts/Mb
MMR	–	pMMR	pMMR	pMMR	pMMR	pMMR	pMMR
MSI	–	MSS	MSS	MSS	MSS	MSS	MSS

*The variant allele fractions (VAFs) and copy numbers of mutated genes were shown. The mutation profiling was obtained by ctDNA or PE-sDNA from the patient. TMB, tumor mutational burden; MMR, DNA mismatch repair; pMMR, proficient MMR; MSI, microsatellite instability; MSS, microsatellite stable*.

* *nonsense mutation*.

## Discussion

Targeted drug therapy has been widely used to treat lung adenocarcinoma (10). The application of next-generation sequencing (NGS) in precision medicine has been approved by FDA to guide clinical drug usage in recent years (11). However, due to tumor heterogeneity and evolvement, drug-resistance becomes an inevitable problem as disease progresses (12). Hence, long-term genetic tests are necessary to monitor the dynamic cancer evolvement to provide precise clinical guidance for the choice of targeted drugs. Another problem arises with the difficulties of obtaining sufficient tumor materials during multiple genetic tests in current practice. In recent years, ctDNA and other alternative minimally invasive approaches, such as PE-sDNA, have been considered as new options to provide sufficient tumor materials (13). In this case report, we report a 71-year-old Chinese male patient with poorly differentiated lung adenocarcinoma, who was treated by a chronological combination treatment of icotinib, osimertinib, and crizotinib, with a series of genetic tests of ctDNA and PE-sDNA at multiple timepoints.

In this case, a chronological combination treatment of icotinib, osimertinib, and crizotinib was chosen due to the mutation of *EGFR* 19del, *EGFR* T790M, and *MET* amplification (14–16). After receiving icotinib therapy, the mutation of *EGFR* 19del was present until the end of the treatment, implying that this patient was not sensitive to icotinib. In contrast, the reduced tumor size and undetectable *EGFR* T790M mutation indicate that the patient responded to osimertinib. After crizotinib treatment, VAFs of *EGFR* 19del, the copy numbers of *MET* amplification, and the level of CEA dramatically decreased, although *MET* amplification still existed in the patient. In addition to *EGFR* 19del and *MET* amplification, *TP53* c.994-1G>C and *FGF23* p.L224^*^, which are not currently actionable by targeted drugs, might lead to final tumor progression.

In this report, VAFs and copy numbers of mutated genes give a hint about the correlation with the physical conditions and traditional biomarkers of patients. Several studies have demonstrated that ctDNA is more sensitive than traditional biomarkers, such as CEA (Carcinoma Embryonic Antigen) (17, 18). However, quantitative comparisons of both VAFs and copy numbers between two separate genetic tests need to be carefully considered to make clinical predictions. This is due to the reason that VAFs and copy numbers of mutated genes are related to the proportion of tumor cells in samples, especially in tissues. Due to the relatively low concentration of ctDNA in blood (19), a maximum threshold of VAFs or copy numbers may also exist. In such circumstance, VAFs or copy numbers of mutated genes in ctDNA may not increase when the tumor progresses. Future studies are needed to elucidate the correlation between the variation of VAFs/copy numbers and physical conditions.

NGS of ctDNA from peripheral blood has allowed the longitudinal monitoring of genetic mutations in a minimally invasive way (20). Additionally, sediment cell genomic DNA (PE-sDNA) from pleural effusion can be used as an alternative minimally invasive approach (21) to detect large scale genetic profiling of the tumors. The dynamics of both ctDNA and PE-sDNA can imply the clinical conditions of the patient by analyzing the VAFs or copy numbers of mutated genes. There are both similarities and differences between ctDNA and PE-sDNA. In this case report, PE-sDNA presented consistent mutated genes with ctDNA, while the VAFs or copy numbers of mutated genes in PE-sDNA were higher than those in ctDNA. This can be explained that the proportion of tumor cells in PE-sDNA is higher compared with ctDNA. However, whether this phenomenon is universal in every individual case is still mysterious. Future investigations are demanded to answer this question. Notably, sample types and sampling methods should be carefully considered when performing analysis and making comparisons based on the VAFs or copy numbers of specific genes.

To our best knowledge, this case report firstly provides insights on the chronological combination of icotinib, osimertinib, and crizotinib. Besides, genomic profiling of ctDNA and PE-sDNA shows the great value for the diagnosis of the patients with lung adenocarcinoma, especially when tumor tissue is not available. Importantly, genetic tests of PE-sDNA can be also applied in other kinds of cancers where PE-sDNA is available, such as breast cancer (22) and ovarian cancer (23). Moreover, a series of genetic tests at multiple timepoints also play an important role, by providing longitudinal monitoring of genetic mutations and guiding the dynamic choices of targeted drugs. Together, these two strategies provide great significance in future clinical practice.

## Conclusion

In summary, with a series of genetic tests of ctDNA and PE-sDNA at multiple timepoints, this case report provides insights on the chronological combination treatment of icotinib, osimertinib, and crizotinib, and demonstrated its potential in developing new strategies for precision medicine.

## Ethics Statement

The studies involving human participants were reviewed and approved by Shangrao Guangxin TCM Hospital Ethical committee. The patients/participants provided their written informed consent to participate in this study.

## Author Contributions

XS and SC conceived and revised the manuscript. ZM designed the clinical treatment for the patient. TM wrote the manuscript. TM, LL, MJ, ZW, HW, CL, and YL performed and analyzed the genetic tests. JR, XG, DoZ, DaZ, and XL performed the clinical treatment for the patient. All authors contributed to the article and approved the submitted version.

## Conflict of Interest

TM, LL, MJ, ZW, HW, CL, YL, and SC were employed by the company, HaploX Biotechnology. The remaining authors declare that the research was conducted in the absence of any commercial or financial relationships that could be construed as a potential conflict of interest.
