# Supplementary material for: Case Report: A Chronological Combination Treatment of Icotinib, Osimertinib, and Crizotinib on Lung Adenocarcinoma Guided by Serial Genetic Tests of Circulating Tumor DNA and Sediment Cell Genomic DNA From Pleural Effusion
Source: Front Oncol. 2020 Oct 23;10:561341. doi: 10.3389/fonc.2020.561341 (PMC7645070; doi:10.3389/fonc.2020.561341)
Supplement: Supplementary file 1 [file Data_Sheet_1.DOCX]

**Supplementary Information 1. Gene list of 605 panel**

#SNV, InDel (464 genes)

| ABL1 | ACTL6A | ACVR1 | ADH1B | AKT1 | AKT2 | AKT3 | ALDH2 | ALK | AMER1 |  |
| --- | --- | --- | --- | --- | --- | --- | --- | --- | --- | --- |
| ANXA5 | APC | AR | ARAF | AREG | ARID1A | ARID1B | ARID2 | ASNS | ASPH |  |
| ASXL1 | ATM | ATR | ATRX | AURKA | AURKB | AXIN1 | AXIN2 | AXL | B2M |  |
| BAP1 | BARD1 | BCL2 | BCL2L1 | BCL2L11 | BCL6 | BCOR | BCORL1 | BLM | BMPR1A |  |
| BRAF | BRCA1 | BRCA2 | BRD2 | BRD4 | BRIP1 | BTK | BUB1 | CACNA1C | CADM2 |  |
| CALR | CAMTA1 | CAPN2 | CARD11 | CASP8 | CBFB | CBL | CBLB | CCL18 | CCND1 |  |
| CCND2 | CCND3 | CCNE1 | CD274 | CD79A | CD79B | CDC73 | CDH1 | CDK12 | CDK4 |  |
| CDK6 | CDK8 | CDKN1A | CDKN1B | CDKN1C | CDKN2A | CDKN2B | CDKN2C | CEBPA | CFD |  |
| CHD4 | CHEK1 | CHEK2 | CIC | CNTNAP5 | CREBBP | CRKL | CRLF2 | CSF1R | CSF3R |  |
| CSMD3 | CTCF | CTNNB1 | CUL3 | CXCR4 | CYLD | CYP19A1 | DAXX | DDIT3 | DDR2 |  |
| DDX3X | DDX51 | DICER1 | DNMT3A | DOT1L | EED | EGFR | ELAC2 | EP300 | EPCAM |  |
| EPHA2 | EPHA3 | EPHA5 | EPHA7 | EPHB1 | ERBB2 | ERBB3 | ERBB4 | ERCC1 | ERCC2 |  |
| ERCC3 | ERCC4 | ERCC5 | EREG | ERG | ERRFI1 | ESR1 | ESR2 | ETV1 | ETV4 |  |
| ETV6 | EWSR1 | EXT1 | EXT2 | EZH2 | FAM175A | FAM46C | FANCA | FANCB | FANCC |  |
| FANCG | FANCI | FANCL | FAT1 | FBN3 | FBXW7 | FGF1 | FGF10 | FGF19 | FGF2 |  |
| FGF23 | FGF3 | FGF4 | FGF5 | FGF6 | FGF7 | FGF8 | FGF9 | FGFR1 | FGFR2 |  |
| FGFR3 | FGFR4 | FH | FLCN | FLT1 | FLT3 | FLT4 | FOXA1 | FOXK2 | FOXL2 |  |
| FOXM1 | FOXP1 | FOXP2 | FUBP1 | FUS | GAB2 | GATA1 | GATA2 | GATA3 | GATA6 |  |
| GEMIN6 | GEN1 | GK5 | GLI1 | GLIPR1 | GLRX | GMEB1 | GNA11 | GNAQ | GNAS |  |
| GPER1 | GPRIN2 | GREM1 | GRIN2A | GSK3B | H3F3A | HDAC2 | HFE2 | HGF | HIF1A |  |
| HMGA2 | HNF1A | HNF1B | HOXB13 | HRAS | HSD17B3 | HSD3B2 | HSP90AA1 | IDH1 | IDH2 |  |
| IFNL2 | IFNLR1 | IGF1R | IGF2 | IKBKE | IKZF1 | IL7R | INHBA | INPP4B | IRF4 |  |
| IRS2 | JAK1 | JAK2 | JAK3 | JUN | KCNJ5 | KDM5A | KDM5C | KDM6A | KDR |  |
| KEAP1 | KIF1B | KIT | KLF4 | KLLN | KMT2A | KMT2B | KMT2C | KMT2D | KRAS |  |
| KRT14 | KRT15 | KRT5 | LARP4 | LATS1 | LATS2 | LBR | LMO1 | LRIG3 | LRP1B |  |
| LYN | MAP2K1 | MAP2K2 | MAP2K4 | MAP3K1 | MAPK1 | MAPK3 | MAPKBP1 | MAX | MCL1 |  |
| MDC1 | MDM2 | MDM4 | MED12 | MEF2B | MEN1 | MET | MITF | MKI67 | MLH1 |  |
| MLH3 | MPL | MRE11A | MSH2 | MSH3 | MSH6 | MST1R | MTOR | MTUS1 | MUTYH | |
| MYC | MYCL | MYCN | MYD88 | MYOD1 | NAB2 | NBN | NCOA3 | NF1 | NF2 | |
| NFE2L2 | NFKBIA | NKX2-1 | NOTCH1 | NOTCH2 | NOTCH3 | NOVA1 | NPM1 | NR4A3 | NRAS | |
| NRG1 | NSD1 | NTRK1 | NTRK2 | NTRK3 | NUP93 | NUTM1 | PAK1 | PALB2 | PALLD | |
| PARK2 | PARP1 | PAX3 | PAX5 | PAX7 | PAX8 | PBRM1 | PCBP1 | PDCD1 | PDCD1LG2 | |
| PDGFB | PDGFRA | PDGFRB | PDPK1 | PGR | PIK3CA | PIK3CB | PIK3CD | PIK3CG | PIK3R1 | |
| PIK3R2 | PIM1 | PLAUR | PLCG2 | PLIN2 | PMS1 | PMS2 | POLD1 | POLE | PPIB | |
| PPP2R1A | PPP2R2A | PRDM1 | PREX2 | PRKACA | PRKACB | PRKAR1A | PRKCI | PRSS1 | PSME2 | |
| PTCH1 | PTEN | PTN | PTPN11 | PTPRD | PTPRT | PZP | RAC1 | RAD21 | RAD50 | |
| RAD51 | RAD51B | RAD51C | RAD51D | RAD52 | RAD54L | RAF1 | RARA | RB1 | RBM10 | |
| RECK | RECQL | RECQL4 | REL | RET | RHBDF2 | RHEB | RHOA | RICTOR | RIF1 | |
| RILP | RIT1 | RNASEL | RNF43 | ROBO2 | ROS1 | RPS6KB1 | RPTOR | RSF1 | RUNX1 | |
| SBDS | SDHA | SDHAF2 | SDHB | SDHC | SDHD | SELL | SETBP1 | SETD2 | SETD7 | |
| SF3B1 | SH2B3 | SHOX | SLX4 | SMAD2 | SMAD3 | SMAD4 | SMARCA4 | SMARCB1 | SMO | |
| SOCS1 | SOCS6 | SOX2 | SOX9 | SPEN | SPINK1 | SPOP | SRC | SRD5A2 | SRSF2 | |
| SS18 | STAG2 | STAT3 | STK11 | SUFU | SULT1A1 | SUZ12 | SYK | SYNE1 | TBX3 | |
| TERT | TET1 | TET2 | TFE3 | TGFBR2 | TMEM127 | TMPRSS2 | TNFAIP3 | TNFRSF14 | TNFRSF19 | |
| TNFSF8 | TOP1 | TP53 | TPMT | TRAF1 | TSC1 | TSC2 | TSHR | TSPAN31 | TYMS | |
| U2AF1 | UGT1A1 | VEGFA | VHL | WAS | WRN | WT1 | XPO1 | YAP1 | YES1 | |
| ZBTB16 | ZNF367 | ZNF717 | ZNF750 |  |  |  |  |  |  | |

#CNV (42 genes)

| ALK | AR | ATM | ATRX | BRCA1 | BRCA2 | CCND1 | CCNE1 | CD274 | CDK4 |
| --- | --- | --- | --- | --- | --- | --- | --- | --- | --- |
| CDKN2 A | CTNNB1 | EGFR | EPCAM | ERBB2 | FGF3 | FGF4 | FGFR1 | FGFR2 | FGFR3 |
| FOXP1 | IKZF1 | JAK2 | KDR | KIT | KRAS | MDM2 | MDM4 | MET | MYCN |
| NOTCH 1 | PDGFRA | PIK3CA | PTEN | RAF1 | REL | RICTOR | SMAD4 | SRC | STK11 |
| TERT | TOP1 |  |  |  |  |  |  |  |  |

#Gene rearrangement (27 genes)

| NTRK1 | PAX7 | | FGFR2 | | RET | KMT2A | | ETV6 | | NAB2 | DDIT3 | | NUTM1 | | FUS |
| --- | --- | --- | --- | --- | --- | --- | --- | --- | --- | --- | --- | --- | --- | --- | --- |
| RARA | SS18 | | PRKACA | | PAX8 | PAX3 | | ALK | | TMPRSS2 | EWSR1 | | PDGFB | | FGFR3 |
| ROS1 | | BRAF | | MYC | | | NRG1 | | NR4A3 | | | NTRK2 | | TFE3 | |

# Predisposing genes (58 genes)

| APC | ATM | | AXIN2 | | BRCA2 | | BARD1 | BLM | BMPR1A | | BRCA1 | | BRIP1 | | CDC73 |
| --- | --- | --- | --- | --- | --- | --- | --- | --- | --- | --- | --- | --- | --- | --- | --- |
| CDH1 | CDK4 | | CDKN1B | | CDKN2A | | CHEK2 | EPCAM | EXT1 | | EXT2 | | FH | | FLCN |
| GREM1 | MAX | | MEN1 | | MET | | MITF | MLH1 | MLH3 | | MRE11A | | MSH2 | | MSH6 |
| MUTYH | NBN | | NF1 | | NF2 | | NTRK1 | PALB2 | PMS1 | | PMS2 | | POLD1 | | POLE |
| PTEN | RAD50 | | RAD51C | | RAD51D | | RB1 | RET | SDHA | | SDHAF2 | | SDHB | | SDHC |
| SDHD | | SMAD4 | | STK11 | | TMEM127 | | TP53 | | TSC1 | | TSC2 | | VHL | |

# Noncoding region gene (3 genes)

| ESR1 | TERT | TERC |
| --- | --- | --- |

# Genes related with targeted drugs and chemotherapies (144 genes)

| MTHFR | CDA | CMPK1 | | IL23R | DPYD | GSTM3 | FCGR2A | FCGR3A | | RGS5 | SELE |
| --- | --- | --- | --- | --- | --- | --- | --- | --- | --- | --- | --- |
| PTGS2 | CFH | EPHX1 | | NCOA1 | GALNT14 | CYP1B1 | MGAT4A | MAP4K4 | | IL1B | LRP2 |
| PARD3B | UGT1A9 | UGT1A6 | | UGT1A4 | UGT1A | OTOS | XPC | SCN10A | | NR1I2 | SLC15A2 |
| ABCC5 | ABCG2 | ADH1C | | CXXC4 | EGF | VEGFC | ADCY2 | PTGER4 | | HMGCR | DHFR |
| XRCC4 | SLC22A4 | SLC22A5 | | IL13 | NQO2 | EDN1 | GPX5 | HLA-G | | TNF | SLC29A1 |
| GSTA1 | SLC22A16 | ECT2L | | OPRM1 | SOD2 | SLC22A1 | SLC22A2 | MAD1L1 | | IGFBP3 | POR |
| SEMA3C | ABCB1 | CYP3A5 | | CYP3A4 | NOS3 | NAT2 | GSR | GGH | | C8orf34 | TNFRSF11B |
| XPA | SLC31A1 | HSPA5 | | PTGES | AKR1C3 | C10orf11 | CYP2C19 | CYP2C8 | | ABCC2 | NT5C2 |
| CASP7 | EIF3A | ARMS2 | | HTRA1 | CYP2E1 | H19 | RRM1 | RRAS2 | | MALAT1 | GSTP1 |
| FOLR3 | DYNC2H1 | WNT5B | | SLCO1B3 | SLCO1B1 | HOTAIR | WIF1 | LGR5 | | E2F7 | TNFSF11 |
| ATP7B | ABCC4 | FNTB | | XRCC3 | SLC28A2 | PIGB | CYP1A1 | CYP1A2 | | IL16 | SLC28A1 |
| UBE2I | RBFOX1 | ABCC1 | | ABCC11 | ZNF423 | NQO1 | ALOX12 | SHMT1 | | LIG3 | C18orf56,TYMS |
| ENOSF1 | CYP2B6 | TGFB1 | | XRCC1 | ACSS2 | TUBB1 | CBR1 | CBR3 | | DSCAM | ABCG1 |
| SLC19A1 | XBP1 | SOX10 | | CYP2D6 | PRDX4 | APLF | UGT1A1 | TPMT | | CCND1 | CYP19A1 |
| BCL2 | | | ERCC2 | | | ERCC1 | | | TYMS | | |

#MSI (5 genes)

| BAT-25 | BAT-26 | NR-21 | NR-24 | NR-27 |
| --- | --- | --- | --- | --- |
